# Sedentary Behaviors and Physical Activity Habits Independently Affect Fat Oxidation in Fasting Conditions and Capillary Glucose Levels After Standardized Glucose-Rich Meal in Healthy Females

**DOI:** 10.3389/fphys.2020.00710

**Published:** 2020-07-07

**Authors:** Sarah A. Tabozzi, Simona G. Di Santo, Flaminia Franchini, Federica Ratto, Matilde Luchi, Beatrice Filiputti, Luca P. Ardigò, Claudio L. Lafortuna

**Affiliations:** ^1^Istituto di Bioimmagini e Fisiologia Molecolare, Consiglio Nazionale delle Ricerche, Segrate, Italy; ^2^Fondazione S. Lucia, Istituto di Ricovero e Cura a Carattere Scientifico, Rome, Italy; ^3^Dipartimento di Neuroscienze, Biomedicina e Movimento, Scuola di Scienze Motorie, Università di Verona, Verona, Italy; ^4^Istituto di Fisiologia Clinica (Sede di Milano), Consiglio Nazionale delle Ricerche, Milan, Italy

**Keywords:** sedentary behavior, moderate-to-vigorous physical activity, lipid metabolism, blood glucose, glucose-rich meal

## Abstract

**Purpose:**

Sedentary behaviors and muscle inactivity are being growingly recognized as important risk factors for health, adjunctively and independently from a scarce physical activity (PA), although the metabolic mechanism underneath is barely clear. To explore the relation between sedentary behaviors (SBs) and metabolism, we measured the metabolic profile in fasting condition and after oral glucose overload in a group of women, along with objective monitoring of their PA/sedentary lifestyle habits.

**Subjects and Methods:**

Thirteen women (age: 32.5 ± 16.1 years; BMI: 24.0 ± 3.3 kg/m^2^), recruited among university students and research staff, underwent indirect calorimetry to assess fat and carbohydrate contribution to energy metabolism, in fasting conditions and after a glucose-rich standard meal (about 45 g of glucose). Glucose concentration in capillary blood was determined in fasting state and 15 and 30 min after meal. Habitual PA and SBs in the previous week were continuously monitored with Actigraph accelerometers.

**Results:**

After adjustment for age, the contribution of fat oxidation to metabolic energy sources, normalized for fat-free mass, in fasting conditions was significantly correlated with time spent in sitting/lying position during wake hours (*p* < 0.001), independent from PA habits, whereas capillary blood peak and change of glucose concentration after the meal were significantly and inversely correlated with average daily moderate to vigorous PA (*p* = 0.025 and *p* = 0.019, respectively), independent from average daily sitting/lying time.

**Conclusions:**

Here, we report for the first time a direct effect of muscle inactivity on increased fat oxidation in fasting conditions, which can be hypothesized as a preliminary condition for the development of insulin resistance. We also report the direct independent effect of PA on the capacity to respond to a glycemic load, so that SBs and reduced PA appear to concur, although independently, to the increased health risk, as elsewhere observed on an epidemiological ground.

## Introduction

The health benefits of physical activity (PA) [defined by World Health Organization (WHO) as “any bodily movement produced by skeletal muscles that requires energy expenditure”] have been fully recognized in the last decades and detailed quantitative recommendations have been globally agreed (World Health Organization, 2010). However, aside from the well-identified health risks deriving from the scarcity of PA, sedentary behaviors (SBs) [defined as “any waking behavior characterized by an energy expenditure (EE) ≤1.5 metabolic equivalents (METs), while in a sitting, reclining or lying posture” (Tremblay et al., 2017)] and muscle inactivity are being growingly recognized to bear an increased risk of fatal and non-fatal cardiovascular diseases and all causes mortality in a dose-dependent way, adjunctively and independently from insufficient PA (Katzmarzyk et al., 2009; Ford and Caspersen, 2012; Biswas et al., 2015). Alarmingly, most of the available data indicate that a considerably large part of adults in westernized countries spend more than 7–8 h per day in the sitting position (Matthews et al., 2008; Loyen et al., 2016). Thus, together with insufficient PA (Troiano et al., 2008) and overconsumption of unhealthy diets with high fat and sugar content (Imamura et al., 2015), widespread SBs concur in characterizing the unhealthy profile of modern societies lifestyle.

Yet, the molecular mechanisms directly involved in the negative effects of muscle inactivity are not completely well understood. In fact, while, on one side, epidemiological studies show that the negative impact deriving from highly sedentary lifestyle is mainly directed toward a dysregulation of glucose homeostasis control and insulin sensitivity (Brocklebank et al., 2015), preclinical studies on rodents, on the other side, evidenced a direct effect of muscle inactivity on lipid metabolism regulation, the activity of lipoprotein lipase (a protein importantly involved in the regulation of triglycerides and high-density lipoprotein metabolism and other metabolic risk factors) being specifically reduced by even relatively short muscle immobilization (Bey and Hamilton, 2003; Hamilton et al., 2004). Indeed, a direct reciprocal interaction between fat and glucose metabolism is well recognized to interfere with the choice of different oxidative energy sources (Randle, 1998) and abnormalities in fat metabolism appear to affect the sensitivity to insulin in skeletal muscles (Koves et al., 2008), so that a complex metabolic interaction between fat and glucose metabolism is involved in the regulation of the homeostatic effect of insulin.

It is remarkable that, in experimental settings, even small changes in sitting time, either on the increase and on the decrease, produce opposite effects on glucose homeostatic system (Lyden et al., 2015; Healy et al., 2017; Winkler et al., 2018), suggesting that muscle inactivity may be associated very tightly to the mechanisms controlling metabolism. So, in view of the large prevalence of sedentary activities in modern societies, a better knowledge of the specific aspects of SBs negatively affecting metabolic health seems to be an important prerequisite to plan adequately targeted behavior change interventions aimed at reducing sitting time and improve health.

Therefore, we hypothesized that individual sedentary habits may affect the fasting metabolic profile and the responses to glucose overload. To test this hypothesis in a small sample of healthy women, we used parameters of SBs and PA (obtained from objective monitoring) as predictors of (a) fat contribution to energy metabolism in fasting conditions and (b) blood glucose levels in response to a test meal.

## Subjects and Methods

### Participants

Thirteen healthy females have been recruited among university nurse students and among research staff. After subjective and objective characterization of their PA and SBs for seven consecutive days, all participants performed a metabolic study of energy sources in fasting conditions, and after a standardized glucose-rich meal.

Exclusion criteria of the participants were presence of cardio-respiratory and/or orthopedic disease, hypertension and/or metabolic disease (diabetes, dyslipidemia), eating disorders, systematic use of drugs for any disease (including oral contraceptives), as well as the habitual practice of agonistic sports activities.

Permission for this study was obtained from the Ethics Committee of Fondazione Santa Lucia, IRCCS, Roma, Italy (for university nurse students) and from Commissione per l’Etica della Ricerca e la Bioetica of Consiglio Nazionale delle Ricerche, Roma, Italy (for research staff). All subjects gave their written informed consent before the measurements.

### Study Design

After recruitment, all subjects underwent an objective characterization of their daily PA and SBs wearing an accelerometric device (Actigraph) for seven consecutive days. The day following the completion of the monitoring, during a specific morning session, each subject performed a metabolic study of energy sources in fasting conditions, immediately followed by a standardized glucose-rich meal and a repetition of the metabolic study.

### Procedures

#### Anthropometrics and Body Composition

The day of the metabolic testing, height, body weight, and composition were measured, respectively, with a stadiometer (mod. 217, Seca, Hamburg, Germany) and a medically approved body composition electronic scale (mod. SC-240 MA, Tanita, Amsterdam, Netherlands); BMI was calculated as body mass/height^2^. The physical characteristics of the participants are summarized in Table 1.

**TABLE 1:** The anthropometric characteristics of participants.

	Age	Body Mass	BMI	Fat Mass	Body Water
	(years)	(kg)	(kg/m^2^)	(%)	(kg)
Mean	32.54	64.80	24.02	30.28	48.42
±SD	16.12	12.84	3.32	7.87	5.78

#### Lifestyle Objective Monitoring

Physical activity and SB were objectively monitored during the 7 days preceding the metabolic testing, using the ActiGraph GT3X + BT (AG; ActiGraph, Pensacola, FL). The device has been chosen because of acknowledged high inter-instrument and intra-instrument reliability in the conditions of common human activities (Santos-Lozano et al., 2012) and because it has been broadly used in research, providing high rates of validity in measuring PA levels (Sasaki et al., 2011). All subjects wore the AG using an elastic waist belt, which kept the sensor just below the iliac crest on the non-dominant side, at the intersection with the axillary line. Waist positioning has been chosen because of demonstrated higher accuracy (Knaier et al., 2019), and because it permits the use of inclinometer function for the detection of posture. The output of inclinometer is the record of the time spent in sitting, reclined and standing positions, over the hours of actual accelerometer functioning and expressed as percent of wear time. Beside the postural monitoring, we also used accelerometer data to characterize daily PA. Participants were asked to wear the accelerometer from the moment they woke up until they went to bed at night and were requested to remove it only during water-based activities such as showering and swimming. Valid days of measurement were considered only for those with ≥8 h of continuous wear. Non-wear time was automatically detected in post-processing using the embedded Choi algorithm (Choi et al., 2011), defining non-wear times as periods of consecutive 0 counts of a certain duration, which is set by default by the manufacturer to 90 min. Data were verified against the activity diary filled out by participants (concerning the end and the beginning of the wear time, day by day). Metabolic intensity was evaluated based on accelerometer counts (Freedson et al., 1998). All waking activities have been stratified into four classes with defined metabolic intensity, labeled as follows: sedentary activity (≤1.5 METs), light-intensity activity (>1.5 to ≤4 METs), moderate-intensity activity (>4 to ≤7 METs), vigorous and very vigorous intensity (>7 METs), and the fraction of time spent performing activities in each class have been recorded and expressed as percent of wear time. In particular, we characterized participants based on their daily moderate and vigorous physical activity (MVPA), and the parameter is relevant for the discrimination of active/non-active, according to international guidelines (World Health Organization, 2010). Along with the intensity of movement, the number of steps per day and the duration of bouts and breaks of sedentary activity have also been considered (Chastin et al., 2015). A sedentary bout was defined as a period of uninterrupted sedentary activity lasting at least 10 min, whereas a sedentary break was defined as the time between sedentary bouts.

#### Metabolic Assessment

In all subjects, the metabolic assessment of energy provided by the oxidation of different substrates was accomplished with open circuit indirect calorimetry, in the morning (measurements starting between 08:00 and 10:00 am), after overnight fasting, using a metabolic system (K5, Cosmed, Italy) with soft silicon facemask. Detailed instructions had been given to participants concerning the overnight fast and the assumption of a regular light meal the evening preceding the test (several examples of menus, based on balanced lipid/carbohydrate and low protein content, had been previously provided to participants). Moreover, they were asked to avoid structured PA, coffee, tea, and alcohol consumption in the 24 h preceding the test. Participants in child-bearing age have been scheduled in order to be tested within the first 2 weeks after the menstrual cycle.

Before each test, the gas analyzers were calibrated using a reference gas mixture (17% O_2_ and 5% CO_2_ in nitrogen). The rate of oxygen consumption (VO_2_) and carbon dioxide production (VCO_2_), standardized for temperature, barometric pressure, and humidity, were recorded at 10-s intervals and the respiratory quotient (RQ) was calculated as RQ = VCO_2_/VO_2_. After about 20 min of acclimatization in the lab facilities, measurements were continuously performed for 30 min, in thermo-neutral environment, while the subjects sat comfortably on an armchair in semi-reclined position, the last 10 min of acquired data being used for analyses. From measured VO_2_ (in L/min), the EE representing the metabolic rate, expressed in kJ/min, was calculated according to the formula given by Garby and Astrup (1987), which accounts for the RQ-dependent variation of O_2_ energy equivalence, as: EE = (4.94 × RQ + 16.04) × VO_2_. The rate of fat oxidation (EE_FOX_), expressed in kJ/min, was calculated by the equation derived by Frayn (1983) and Lazzer et al. (2010), assuming as negligible the contribution of protein oxidation in resting conditions: EE_FOX_ (kJ/min) = (1.67 × VO_2_ (L/min) − 1.67 × VO_2_ (L/min) × RQ) × 37.66. Conversely, the rate of carbohydrate oxidation (EE_*COX*_) was calculated as: EE_*COX*_ (kJ/min) = (4.55 × VO_2_ (L/min) × RQ - 3.21 × VO_2_ (L/min)) × 16.74. The percentage of FOX (FOX%) was calculated as: FOX% = EE_FOX_/(EE_FOX_ + EE_*COX*_) × 100. In order to account for the effect of body composition in the analysis of results, the individual values of EE_FOX_ were normalized for the amount of fat-free mass (EE_FOX_/FFM).

The test in fasting conditions was immediately followed by a standardized glucose-rich meal, consisting of 6 biscuits, 30 g of apricot jam and 200 ml of fruit juice, which overall contained about 45 g of glucose. A second 30-min metabolic test started immediately after the meal and was conducted with the same procedure as the previous one, and similarly analyzed. Capillary blood samples were obtained from a fingertip before the meal, and 15 and 30 min thereafter, for the measurement of glucose concentration (G-Sensor, Menarini, Italy). A preliminary blood sampling performed on a small group of eight different women with similar characteristics at 15-min interval up to 60 min after a similar test meal had revealed that peak glucose concentration was systematically detected between the 15th and the 30th min after the completion of the meal.

### Statistical Analysis

All numerical data are reported as mean value ± standard deviation from the mean (SD). Significance of differences between mean values measured in fasting condition and those measured after the test meal was assessed with a student’s *t* test for paired measurements. Correlations between metabolic outcomes and PA/SB indicators were evaluated with univariate Spearman rho coefficients. A multiple regression analysis was used to test the independent effect of PA and SB on the relevant metabolic parameters, controlling for the effect of age. *P* values <0.05 were considered statistically significant.

## Results

Table 2 shows the accelerometry-based profile of PA and SB obtained in the participants of the study throughout the week preceding the metabolic measurements.

**TABLE 2:** The profile of physical activity and sedentary behavior obtained in the participants of the study, referred to the week preceding the metabolic measurements.

	Days wear	Average wear time per day	Sitting and reclined position^†^	Sedentary activity^†^	Light intensity activity^†^	Moderate intensity activity^†^	Vigorous intensity activity^†^	MVPA per day	Steps per day	Sedentary bouts per day	Length of sedentary bouts	Length of sedentary breaks
	–	h:m:s	%	%	%	%	%	min	*n*	*n*	min	min
**Objective assessment with Actigraph**												
Mean	6.1	12:49:56	68.5	75.2	19.1	5.3	0.30	45.9	7866	11.9	16.6	101.7
±SD	0.9	01:20:40	9.5	3.5	2.6	1.8	0.64	17.2	2059	1.5	1.5	18.6
Min	5	10:47:33	52.5	69.0	15.2	1.6	0.00	14.8	4995	9.7	13.9	82.1
Max	8	15:18:24	83.3	80.4	23.2	7.8	2.39	79.4	12233	14.0	19.5	151.4

*^†^Values considered as average% of daily wear time. MVPA, moderate to vigorous physical activity.*

The average value of metabolic parameters measured in fasting conditions and after the glucose-rich meal are summarized in Table 3. After the test meal, due to the thermogenic effect of the food, a significant increase was observed in oxygen uptake and EE, while the significant increase in RQ corresponded to a significant reduction of fat utilization as energy source, in the presence of a rise of about 65% of glucose concentration in capillary blood.

**TABLE 3:** Metabolic parameters and glucose concentration in capillary blood measured at rest in fasting conditions and after a standardized glucose-rich meal.

		Fasting	After load	*P* (paired *t* test)
VO_2_	ml/min/kg	4.29 ± 0.65	4.76 ± 0.61	0.005
EE	kJ/min	5.41 ± 0.96	6.16 ± 0.81	0.001
RQ	–	0.756 ± 0.029	0.866 ± 0.035	<0.001
EE_FOX_/FFM	J/min/kg	93.8 ± 15.3	58.2 ± 17.9	<0.001
FOX%	%	79.7 ± 11.2	41.0 ± 11.7	<0.001
GLC	mg/dl	90.1 ± 6.7	149.5 ± 27.0	<0.001

*VO_2_, oxygen consumption; EE, energy expenditure; RQ, respiratory quotient; EE_*FOX*_/FFM, energy derived from fat oxidation, expressed per unit fat-free mass; FOX%, fat oxidation, expressed as % of total energy metabolic sources; GLC, glucose concentration in capillary blood (the value after meal is referred to the peak concentration measured either 15 or 30 min after the meal).*

Due to a non-linear distribution of some Actigraph-derived parameters, a preliminary analysis of correlation was performed using a non-parametric test (Spearman rho test). In Table 4, Spearman’s correlation coefficients between variables related to PA and SB and metabolic parameters, obtained in fasting conditions and after the test meal, are presented. Low fasting RQ (and hence a higher FOX% and EE_FOX_/FFM) appears to be primarily correlated with time spent in sitting or reclined position during wake, but not with any other parameter related to PA. By contrast, the amount of at least moderate PA are associated to lower peaks (and swings, ΔGLC) of glucose concentration in capillary blood after the test of glucose-rich meal, without any correlation with sedentary parameters.

**TABLE 4:** Spearman’s univariate correlation coefficients between sedentary behavior/physical activity variables objectively evaluated with Actigraph objectively evaluated with Actigraph and metabolic parameters and metabolic parameters obtained in fasting conditions and after a glucose-rich meal in the subjects of the study (*N* = 13).

	Fasting					After glucose load					
	VO_2_	RQ	FOX%	EE_FOX_/FFM	GLC	VO_2_	RQ	FOX%	EE_FOX_/FFM	GLC	ΔGLC
Age	−0.568*	0.141	–0.088	−0.589*	0.390	–0.399	0.274	–0.217	–0.217	0.317	0.327
Sitting and reclined position^†^	–0.085	−0.734**	0.655*	0.808**	0.284	–0.484	–0.391	0.399	0.302	0.226	0.231
Sedentary activity^†^	0.498	–0.298	0.264	0.626*	0.143	0.140	0.105	–0.088	–0.192	0.297	0.231
Light intensity activity^†^	−0.597*	0.190	–0.165	−0.621*	0.063	–0.206	0.071	–0.102	0.011	0.171	0.264
Moderate intensity activity^†^	–0.149	0.272	–0.162	–0.220	–0.463	–0.146	–0.294	0.317	0.341	−0.762**	−0.714**
Vigorous intensity activity^†^	0.190	0.215	–0.195	–0.016	–0.242	0.374	–0.294	0.320	0.423	−0.569*	−0.592*
MVPA per day	–0.206	0.306	0.198	–0.324	–0.364	–0.124	–0.124	0.182	0.258	−0.707**	−0.661*
Steps per day	–0.374	0.397	–0.283	–0.451	–0.187	–0.195	–0.099	0.083	0.148	–0.492	–0.452
Sedentary bouts per day	0.072	–0.204	0.259	0.104	0.275	–0.124	–0.045	0.019	–0.099	0.135	0.096
Length of sedentary bouts	0.003	–0.233	0.141	0.140	–0.293	0.146	–0.360	0.293	0.240	–0.077	–0.080
Length of sedentary breaks	–0.011	0.130	–0.228	0.033	–0.388	0.135	–0.054	0.105	0.264	–0.300	–0.237

^†^Values considered as average % of daily wear time. VO_2_, oxygen consumption; RQ, respiratory quotient; EE_*FOX*_/FFM, energy derived from fat oxidation, expressed per unit fat-free mass; FOX%, fat oxidation (% of total energy metabolic sources); GLC, glucose concentration in capillary blood (the load value is referred to the peak concentration measured either 15 or 30 min after the meal). ΔGLC, changes in glucose concentration in capillary blood, after the glucose-rich test meal. Significance of correlation (2-tailed): * at the 0.05 level, ** at the 0.01 level.

**FIGURE 1:**
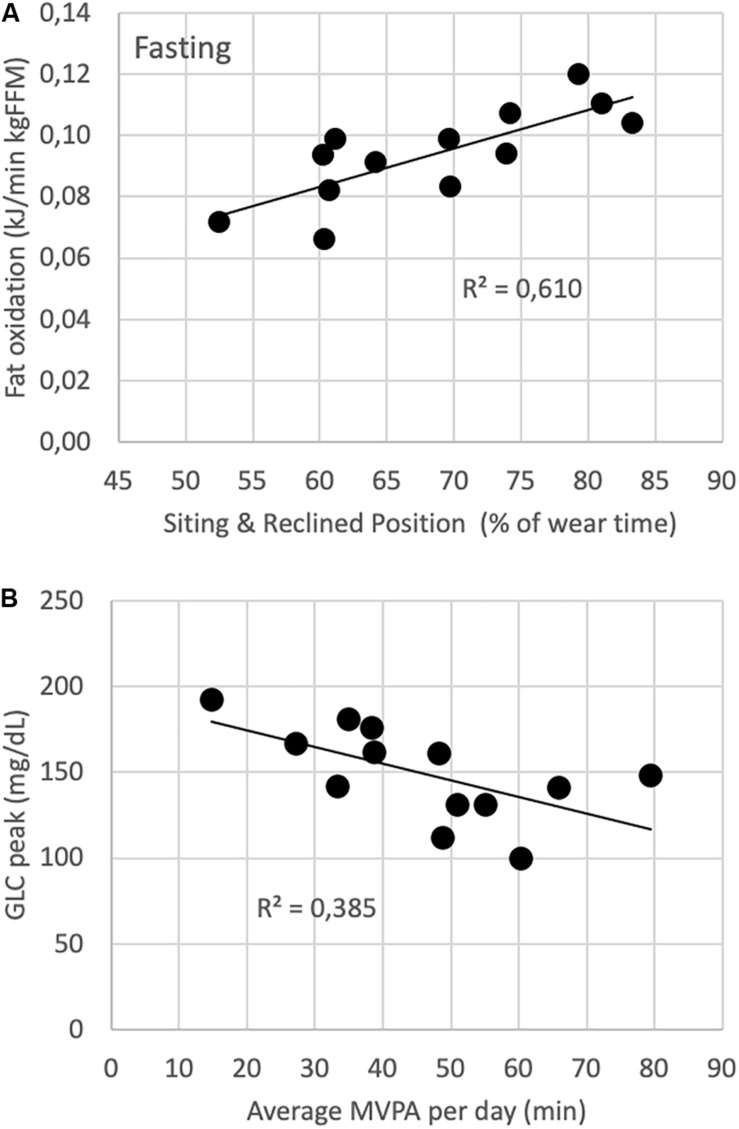
The relationship between sitting/lying time and fat contribution to energy metabolism per unit FFM **(A)**, and between average time of daily MVPA and peak capillary blood glucose level in response to a test meal **(B)**.

Figure 1 shows the relationship between sitting/lying time and fat contribution to energy metabolism per unit FFM (panel A), and between average time of daily MVPA and peak capillary blood glucose level in response to a test meal (panel B).

To better discriminate between the negative effect of sedentary activities and that of the scarcity of PA, a multiple linear regression has been performed, using markers of SB and PA as independent variables and the metabolic outcomes as dependent variables, with age being used as a covariate. Table 5 shows the results of the multiple regression analysis testing the percent of wake time spent in sitting or reclined position (marker of sedentariness) and the average time of daily MVPA (marker of physical activity) as predictors of fasting fat contribution to energy metabolism per unit FFM (EE_FOX_/FFM), and of peak and changes of capillary blood glucose levels in response to oral meal (Load GLC and ΔGLC).

**TABLE 5:** Correlation coefficients obtained using a multiple regression model (controlling for the confounding effect of age) to test the significance of SB and PA parameters as predictors of fat contribution to energy metabolism (EE_FOX_/FFM) and of peak and changes of capillary blood glucose levels in response to oral meal (Load GLC and ΔGLC), in the subjects of the study (*N* = 13).

	Standardized coefficients	*t*	Predictors significance	Model *R*^2^	Model significance
**Dependent variable**					
EE_FOX_/FFM				0.872	<0.001
**Model predictors**					
Age	–0.489	–4.058	0.003		
Sitting and Reclined Position†	0.916	6.62	< 0.001		
MVPA per day	0.281	2.015	0.075		
**Dependent variable**					
Load GLC				0.551	0.05
**Model predictors**					
Age	0.413	1.827	0.101		
Sitting and Reclined Position†	–0.052	–0.200	0.846		
MVPA per day	–0.703	–2.686	0.025		
**Dependent variable**					
ΔGLC				0.548	0.05
**Model predictors**					
Age	0.470	2.073	0.068		
Sitting and Reclined Position^†^	–0.321	–1.233	0.249		
MVPA per day	–0.748	–2.847	0.019		

*EE_*FOX*_/FFM, energy derived from fat oxidation, expressed per unit fat-free mass; Load GLC, glucose concentration in capillary blood referred to the peak concentration measured either 15 or 30 min after the meal; ΔGLC, changes glucose concentration in capillary blood, after the glucose-rich test meal. ^†^Values considered as average% of daily wear time.*

The results confirm that the contribution of lipid oxidation to fasting metabolic requirements is significantly influenced by the amount of time spent in sitting or reclined position, without any effect of the concomitant PA performed, while blood glucose concentration in response to a glucose-rich meal is significantly affected by the amount of daily MVPA, without any effect of sitting/reclined time.

## Discussion

The present study shows the effect of sitting time on fasting metabolic energy sources, independent from PA habits, and the effect of PA on blood glucose peak after a sugar-rich meal, independent from the sitting time, in a small group of women with sedentary occupation.

In spite of an occupational sedentary profile (as that pertaining to university students and research professionals), objective assessment with Actigraph ascertained that, on average, the participants were largely compliant with the recommended levels of PA for adults (Table 2). In fact, according to guidelines released by WHO and other authoritative institutions (European Commission, 2007; World Health Organization, 2010; U. S. Department of Health and Human Services, 2018), only 15% of participants of the study failed to meet the recommended amount of MVPA (which corresponds to at least 150 min of moderate-intensity aerobic PA or 75 min of vigorous-intensity aerobic PA per week, or an equivalent combination of the two). However, our subjects were also on average exceedingly sedentary when compared with available population studies, both in Europe and US (Matthews et al., 2008; Van Dyck et al., 2015), possibly suggesting that individuals occupationally mainly involved in sedentary jobs (like university students and research professionals presently investigated) may have a considerably larger amount of sitting time than the general population enrolled in large cross-sectional national studies.

Indeed, in this study, we objectively assessed both the sit/lying time and the time spent in metabolically sedentary activities (i.e., involving EE ≤ 1.5 METs), which became significantly longer (paired *t* test, *p* < 0.01), with an average difference of about 88 min. This may indicate that slightly more than 1 h per day is spent for activities involving very low metabolic intensity but performed in position not recognized as sitting or lying.

Thus, within the range observed in the subjects of the present study (i.e., 53–83% of average daily wear time), the percent of sitting/lying time (but not the metabolically sedentary time) was a significant predictor of EE_FOX_/FFM in fasting conditions, when an age-adjusted multiple regression model including also daily amount of MVPA was applied (Table 5). The role of sitting and consequent muscle inactivity, more than the low energy required by the metabolically defined sedentary activities, is a central finding of the present study, which overlaps the results of Katzmarzyk (2014), who reported a significant reduction of mortality rate induced by simple standing in the physically inactive group of a large population cohort of adults. Thus, even if sitting and standing have a similarly low energy requirement, they may represent different physiological states with potentially different impact on health (Hamilton et al., 2007).

From the epidemiological standpoint, growing knowledge is robustly providing evidence that SB is in general associated with increased risk of fatal and non-fatal cardiovascular diseases, distinct and independent from that deriving from a low amount of PA (Katzmarzyk et al., 2009; Ford and Caspersen, 2012; Biswas et al., 2015). More specifically, the direct effect of sitting time has been investigated, experimentally testing the partial substitution of sitting with standing, during the work hours (Healy et al., 2017; Winkler et al., 2018). After the reduction of sitting time, small but significant health benefits such as the reduction of blood fasting glucose and cardiometabolic risk scores, on the long term, were detected. Similarly, Aadahl et al. (2014) explored, on a sedentary sample from a Danish population-based study, the effect of an individualized behavior change intervention on sitting time (objectively measured with the positional sensor ActivPal). They found that even a very small (and not significant) reduction of sitting time of about half an hour was effective in reducing significantly fasting serum insulin and insulin resistance markers. Comparable results were obtained by Edwardson et al. (2017) with a cross-sectional analysis using an iso-temporal substitution model in a large group of individuals considered at risk of impaired glucose tolerance or diabetes and a 7-day objective sitting/standing/stepping monitoring with ActivPal; they concluded that a model of reallocation of prolonged sitting bouts to shorter bouts or standing produces significant increase of insulin sensitivity and reduction of blood fasting insulin levels after a 2-h glucose loading test. In agreement with the effect of daily sitting time reduction, even a modest voluntary increase in muscle inactivity (about 15%), deriving from prolonged sitting during leisure time in a 7-day test performed by a small group of recreationally active individuals in their free-living environment, produced significant elevation of the insulin response to a glucose load, without remarkable differences in blood glucose (Lyden et al., 2015). Thus, all these previous findings overall indicate that even small changes in sitting time (in physiological terms likely corresponding to changes in muscle inactivity) have a detectable impact on the hormonal systems involved in the regulation of glucose homeostasis and support the notion that a behavior change approach targeting sedentary habits may provide a relevant benefit for health.

Indeed, P. J. Randle outlined a basic biochemical mechanism that regulates fuel selection in body tissues, based on evidence that cardiac and skeletal muscle can readily shift from carbohydrate to fat and vice versa as energy oxidative sources, depending primarily on their availability, so that oxidation of one nutrient would inhibit the use of the other (Randle, 1995; Kelley and Mandarino, 2000; Hue and Taegtmeyer, 2009). Stemming from this general principle, a specific paradigm for the genesis of insulin resistance has been explored by Koves et al. (2008). Based on an alimentary contest of overabundant diet constantly enriched in both carbohydrates and lipids, unmatched to the low requirements of physical inactivity (which appear to be typical features of common lifestyle in modern societies), the authors suggested that excessively elevated (rather than deficient) fatty acid oxidation, exceeding the tricarboxylic acid cycle capacity, leads to incomplete fat oxidation and mitochondrial stress, ultimately being the obligate prerequisites for the subsequent development of insulin resistance and diabetes (Muoio and Newgard, 2008). Here we report for the first time a direct effect of muscle inactivity, objectively assessed as sitting time during everyday life, on increased fat oxidation in fasting conditions. This may provide the mechanistic basis of the epidemiological observation concerning the negative impact of SBs on health, even if no molecular background is explored. Although insulin has not been measured in the present study, it is remarkable that we failed to detect any effect of sitting time on the metabolic response to a glucose-rich standard meal, likely suggesting that SBs may have no significant direct effect *per se* on glucose metabolism or overt insulin resistance.

However, on the other side, we also observed a significant correlation between daily minutes of MVPA objectively assessed and the smaller peaks (or swings) of glucose concentration in capillary blood after a standardized meal, possibly deriving from an exercise-dependent increase of muscle glucose uptake. Epidemiological evidences indicate that even single bouts of moderate-to-vigorous PA can acutely improve insulin sensitivity over 50% for up to 72 h in non-diabetic individuals (Bird and Hawley, 2017), likely acting through an increase of translocation and expression of GLUT4 induced by muscle contraction (Richter and Hargreaves, 2013). Moreover, an independent effect of PA on insulin sensitivity and sedentary time on intracellular markers of lipid metabolism has been recently reported from a study investigating a very large cohort of healthy individuals using objective assessment of lifestyle behaviors (Spartano et al., 2017). Based on mutually exclusive binary categorization of PA habits and SBs, similar results have been obtained from a large sample of participants of the Health Survey for England 2008 by Bakrania et al. (2016), who evidenced the negative effect of high sedentary time on lipid metabolism (lower HDL cholesterol) independent from the better health profile associated with high levels of PA. Thus, in accordance with these results, the present study confirms that muscle inactivity and PA may affect different metabolic targets and pinpoints the complex and reciprocal interaction between lipid and glucose metabolism.

In conclusion, according to the paradigm of muscle mitochondria overload deriving from an excessive incomplete fat oxidation as a preliminary condition for the development of insulin resistance and diabetes (Koves et al., 2008; Muoio and Newgard, 2008), present data, reporting an association between sitting time and increased use of lipid substrate for energy metabolism, support a conceptual model in which muscle inactivity (in association with a possibly overabundant diet) may contribute to the progression toward insulin resistance. At the same time, the amount of PA daily performed was a significant correlate of the capability to reduce blood glucose peaks after oral glucose load, confirming the important role of exercise in the modulation of the glucose homeostasis system. While widely recognized recommendations provide well-defined guidelines for PA, present results further highlight the urgent need to agree detailed recommendations concerning sedentary activities, which may in practice guide adequate programs for targeted behavior change.

However, due to a number of limitations, such as the relative scarcity of subjects, the lack of measurement of insulin, and protein contribution to energy metabolism, present results should be taken with due caution and considered as preliminary data that need confirmation from a more in-depth investigation considering individuals of different sex through the ages of life, in different health conditions.

## Data Availability Statement

The datasets generated for this study are available on request to the corresponding author.

## Ethics Statement

The studies involving human participants were reviewed and approved by - Ethics Committee of Fondazione Santa Lucia, IRCCS, Roma, Italy and - Commissione per l’Etica della Ricerca e la Bioetica of Consiglio Nazionale delle Ricerche, Roma, Italy. The patients/participants provided their written informed consent to participate in this study.

## Author Contributions

ST conceived and designed the study, recruited subjects (research staff), conducted experiments, analyzed data, and drafted the manuscript. SD supervised recruitment of subjects (university students) and participated in designing the study. FF, FR, ML, and BF participated in conducting experiments and analyzing data. LA participated in editing the manuscript. CL conceived and designed the study, contributed in analyzing data, and edited the manuscript. All authors read and approved the manuscript.

## Conflict of Interest

The authors declare that the research was conducted in the absence of any commercial or financial relationships that could be construed as a potential conflict of interest.
